# Cocaine-Induced Giant Bullous Emphysema

**DOI:** 10.1155/2020/6410327

**Published:** 2020-05-02

**Authors:** Steven Douedi, Vandan D. Upadhyaya, Ishan Patel, Usman Mazahir, Eric Costanzo, Mohammad A. Hossain

**Affiliations:** ^1^Department of Medicine, Jersey Shore University Medical Center, Hackensack Meridian Health, Neptune, NJ 07753, USA; ^2^Department of Pulmonary and Critical Care Medicine, Jersey Shore University Medical Center, Hackensack Meridian Health, Neptune, NJ 07753, USA

## Abstract

**Background:**

Emphysematous bullae, defined as airspaces of greater than or equal to one centimeter in diameter, have a variety of etiologies such as tobacco use and alpha-1 antitrypsin being the most common. Emphysematous bullae have also been reported in patients using cocaine usually involving the lung periphery and sparing the central lung parenchyma. We present a case of a male with a history of cocaine abuse found to have a singular giant emphysematous bulla occupying >95% of the right hemithorax requiring video-assisted thoracic surgery (VATS) with a favorable outcome. *Case Presentation*. A 50-year-old male with a history of chronic cocaine abuse was found unresponsive in the field and given multiple doses of naloxone without any improvement in mental status. On presentation to the emergency department, chest X-ray as well as CT scan of the chest were performed which were suggestive of an extensive pneumothorax of the right lung requiring placement of a chest tube. The patient was subsequently intubated and underwent bronchoscopy with right chest VATS which found a giant bulla encasing the entire right pleural cavity. During the procedure, he underwent resection of the bullae and a partial right pleurodesis. After the procedure, patient's respiratory status significantly improved, and he was discharged in a stable condition.

**Conclusion:**

Cocaine use is a rare but identifiable factor that can cause giant bullous emphysema (GBE) resulting in severe complications and even death. The purpose of this case presentation is to support early identification and treatment of GBE using bullectomy with VATS, improving outcomes and decreasing morbidity and mortality.

## 1. Introduction

Giant bullous emphysema (GBE) is first defined by Roberts et al. as bullae radiographically occupying greater than 30% of one or both hemithorax without compression of surrounding lung parenchyma [1]. GBE is usually due to the enlargement of distal airspaces and terminal bronchioles with alveolar wall destruction [2]. The most common causes of bullous emphysema (BE) include chronic obstructive pulmonary disease (COPD) caused by tobacco use, alpha-1 antitrypsin deficiency [3], and rarely, illicit substance use such as cocaine [4, 5]. GBE due to illicit drug use usually spares the central lung parenchyma and is commonly seen in the upper lung fields and periphery [6]. We present a case of a patient with GBE occupying >95% of the right lung hemithorax caused by chronic cocaine use. Initially believed to be a right-sided pneumothorax that did not resolve with chest tube placement, video-assisted thoracic surgery (VATS) was ultimately performed with successful results as previously described in the literature [7, 8].

## 2. Case Presentation

**Figure 1:**
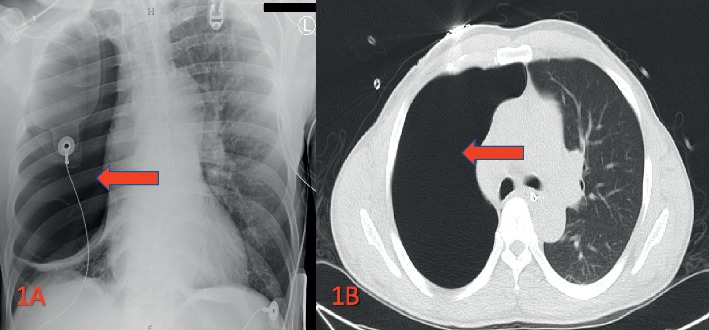
(a) Portable chest X-ray on admission showed extensive lucency in the right hemithorax which suggested large pneumothorax or an air-filled cyst. (b) CT scan of the chest showing one large GBE of the right hemithorax without a mediastinal shift.

A 50-year-old male with a past medical history of chronic cocaine abuse was found unresponsive in the field. He was given multiple doses of naloxone in the field without any improvement in mental status. On presentation in the emergency department (ED), the patient was found unresponsive to questions and unable to follow commands but withdrawing to painful stimuli, with a Glasgow Coma Score of 8, and tachypneic. Vital signs were significant for a respiratory rate of 38 breaths per minute and oxygen saturation of 90% on 6 liters nasal cannula. Physical exam appreciated no tracheal deviation and no jugular venous distention but exhibited an uneven rise and fall of his chest on the right side compared to the left. On cardiopulmonary auscultation, absent breath sounds of the right lung fields were noted with normal cardiac heart sounds. The patient was emergently intubated for airway protection in the ED, and further evaluation was initiated. Immediate imaging with portable chest X-ray was obtained which showed extensive lucency in the right hemithorax suggestive of large pneumothorax or an air-filled cyst (Figure 1(a)). A CT scan of the chest was performed which suggested extensive pneumothorax of the right lung without a mediastinal shift (Figure 1(b)).

**Figure 2:**
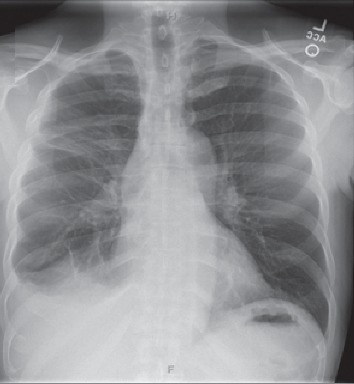
Postprocedure X-ray prior to discharge from the hospital showing improvement of bullae and expansion of the right lung.

For this reason, an emergent 24 French chest tube was placed in the right 6th intercostal space but failed to expand the lung. In response, the chest tube was then exchanged for a 32 French tube under the assumption that a larger size may be required, but it too failed to expand the lung. There was no air drainage from either tube placed suggesting lack of a pneumothorax. He was stabilized and admitted to the intensive care unit for further management. A few hours after admission, he underwent bronchoscopy with a right chest VATS which revealed a giant bulla encasing the entire right pleural cavity with its origin in the right upper lobe beginning at the apex. The bulla was extracted, and pleurodesis was performed by mechanical and chemical means. After the procedure, the patient's respiratory status significantly improved. His partial pressure of oxygen on arterial blood gas was 105 on a fraction of inspired oxygen of 40% and positive end-expiratory pressure of 5. He had excellent ventilatory weaning parameters and was finally extubated to nasal cannula on day 6 of hospitalization. He was transferred to the general medical floors the following day and discharged without supplemental oxygen requirement in a stable condition on day 8.  Figure 2 shows post-VATS chest X-ray at discharge. The patient was evaluated for various causes of GBE while inpatient. Alpha-1 antitrypsin levels were within normal limits, and HIV was not detected. Evaluation of malignancies and cystic lung diseases yielded negative results. Urine drug screen was positive for cocaine, and the patient endorsed no tobacco smoking history and a long history of intravenous cocaine use as well as intranasal prior to discharge. On outpatient follow-up 2 weeks after discharge, the patient remained symptom-free and in good health.

## 3. Discussion

Cocaine is one of the leading causes of drug abuse-related deaths [9]. It has been studied that cocaine destroys the alveolar wall typically in the upper airways. High-resolution computed tomographic (HRCT) findings of patients with cocaine abuse have reported a variety of pulmonary findings—“crack lung,” cardiogenic and noncardiogenic edema, alveolar hemorrhage, interstitial disease, pulmonary hypertension, emphysema, and eosinophilic disease being a few [10, 11]. Cocaine is a rare cause of emphysema occurring in only about 2–4% of intravenous illicit drug abusers; however, when found, it is usually centrilobular and encountered in the periphery of the lungs sparing the central region [6, 12]. Giant bullous emphysema, defined as a region involving greater than 30% of the hemithorax, is another finding appreciated in cocaine users [4]. The bullae are suspected to be the result of alveolar weakness and parenchymal remodeling due to vasoconstriction caused by cocaine inhalation and increase in size due to the elastic recoil of the lung tissue [13].

The patient we describe in this report presented with a unique finding of a singular bulla that had encapsulated the entire right pleural cavity in an intravenous and intranasal cocaine abuser. While the placement of a chest tube showed little to no improvement in this patient's condition, VATS showed marked utility in the treatment of GBE in both literatures review and the case we presented [7, 8]. GBE bullectomy has shown >89% survival rates in a 5-year follow-up study of 43 patients [14]. A large case series investigating users of illicit intravenous drug use found the incidence of bullous pulmonary damage to be only 2% [12]. Almeida et al. have described a case series of HRCT scans of the chest in 22 patients with cocaine-induced pulmonary changes, only 2 of which with findings of bullous emphysema and none of whom were identified with giant bullous emphysema [10]. Due to its rare occurrence, cocaine-induced giant bullous emphysema is often confused for a possible underlying pneumothorax. Imaging studies should be performed in patient's suspected with bullae as chest tube placement has been associated with high flow fistulas leading to worse outcomes [15]. The rapid identification using imaging modalities and immediate treatment for bullous emphysema can lead to improved patient outcomes and decreased morbidity and mortality.

## 4. Conclusion

Cocaine use is a rare but identifiable factor that can cause GBE resulting in severe complications and even death. Usually affecting the upper airways and lung periphery, cocaine can lead to extensive large bullae in one or both hemithorax. As seen in this case presentation, early identification and treatment of GBE using bullectomy with VATS prove to be effective and supports improved patient outcomes and survival. Clinicians should be vigilant and consider GBE as a possibility of respiratory distress while evaluating and treating a patient with a known history of cocaine abuse.
